# Association of Salt Intake with Muscle Strength and Physical Performance in Middle-Aged to Older Chinese: The Guangzhou Biobank Cohort Study

**DOI:** 10.3390/nu15030516

**Published:** 2023-01-19

**Authors:** Tingyu Lu, Weisen Zhang, Chaoqiang Jiang, Yali Jin, Tong Zhu, Feng Zhu, Lin Xu

**Affiliations:** 1 School of Public Health, Sun Yat-sen University, Guangzhou 510080, China; 2 Molecular Epidemiology Research Center, Guangzhou Twelfth People’s Hospital, Guangzhou 510620, China; 3 Institute of Applied Health Research, University of Birmingham, Birmingham B15 2TT, UK; 4 School of Public Health, The University of Hong Kong, Hong Kong, China

**Keywords:** salt intake, grip strength, timed up-and-go test, falls, older people

## Abstract

Older people have higher amounts of sodium accumulation in skeletal muscles than younger people, indicating the possible role of salt intake on muscular and physical function. This large population-based cross-sectional study examined the association of salt intake with muscle strength and physical performance in 4867 participants with an average age of 60.4 (standard deviation = 7.7) years. Information on salt intake was collected from self-reports. Absolute and relative grip strength (AGS and RGS), timed up-and-go test (TUGT), and falls were considered the indicators of muscle strength and physical performance. Linear and logistic regression were used to examine the associations of salt intake with AGS, RGS, TUGT score, and falls, adjusting for demographic and lifestyle factors, body mass index, self-rated health, and self-reported hypertension. Higher salt intake was independently associated with lower grip strength and a higher TUGT score. Versus light salt intake, the adjusted β (95% confidence interval (CI)) of AGS_max_, RGS_max_, and TUGT scores in those with salty taste were −0.53 (−0.97, −0.08) kg, −0.04 (−0.06, −0.02) kg per kg/m^2^, and 0.08 (0.02, 0.14) s, respectively. A non-significant association was found between salt intake and falls. In sex-stratification analysis, the association remained in women but became non-significant in men. Our results suggest that avoiding high-salt diets may play a role in preserving muscle strength and physical function in the elderly, especially in women.

## 1. Introduction

Skeletal muscle aging is a major cause of frailty and disability in the elderly [1] with the loss of muscle mass, muscle strength, and the decline of physical performance [2]. Skeletal muscle aging is a consequence of an unhealthy lifestyle and comorbidities [2]. Lifestyle factors related to muscle strength and physical performance included physical activity [3] and a nutritionally adequate dietary pattern (e.g., sufficient protein and Vitamin D intake) [4,5,6].

High salt intake was deeply rooted in traditional Chinese dietary patterns. According to the national survey carried out from 2009 to 2011, the mean daily salt intake of Chinese was 9.1 g/day [7], which was significantly higher than the recommended amount from the World Health Organization (WHO) (<5 g/day) [8]. It was clear that high salt intake was associated with a high risk of multiple chronic diseases, including hypertension [9] and stroke [10]. One study reported a positive correlation between high sodium intake and high frailty risk, accompanied by poor grip strength and physical function [11]. Since the elderly tend to accumulate more sodium in tissues than younger people [12], higher salt intake may lead to lower muscular and physical function. However, evidence on the direct association of salt intake with muscle strength and physical performance was relatively limited. Up to December 2020, the regularly updated systematic review of salt and health outcomes studies from The World Hypertension League Science of Salt [13] reported only one study exploring the association between sodium intake and physical function [14]. This study showed a negative association between sodium intake and physical performance, as indicated by the Short Physical Performance Battery (SPPB) score [14]. 

Grip strength was an important marker of muscle strength in older people [15,16]. Previous studies with small sample sizes reported a negative association between salt intake and grip strength [17,18]. The timed up-and-go test (TUGT) is another useful tool to assess physical performance, including lower limb strength, balance, and gait in older adults [19,20]. However, to date, we have found no study reporting the association of salt intake with TUGT score. 

Given the inevitable skeletal muscle aging and the stubbornly high salt intake in older Chinese, this study set up to assess the association of salt intake with muscle strength and physical performance, using data from a well-established population-based study, the Guangzhou Biobank Cohort Study (GBCS).

## 2. Materials and Methods

### 2.1. Study Sample

All participants were recruited from the GBCS. Details of the GBCS have been reported previously [21]. Briefly, the GBCS is a 3-way collaboration among the Guangzhou Twelfth People’s Hospital and the Universities of Hong Kong, China, and Birmingham, UK. Participants of the GBCS were recruited from the “Guangzhou Health and Happiness Association for the Respectable Elders (GHHARE)”, a community social and welfare organization. Membership is open to Guangzhou permanent residents age 50 or older for a nominal fee of 4 CNY (≈50 US cents) per month. The baseline examination included a face-to-face, computer-assisted interview by trained nurses to collect information on demographic characteristics, lifestyle factors, family, and personal medical history. Anthropometric parameters, blood pressure, fasting plasma glucose, lipids, and inflammatory markers were measured. The reliability of the questionnaire was tested by recalling 200 randomly selected participants for re-interview, and the results were satisfactory [21]. The Guangzhou Medical Ethics Committee of the Chinese Medical Association approved the study, and all participants gave written, informed consent before participation. In this study, we used the third phase of baseline data from GBCS from September 2006 to September 2007. Participants with cancer or severe cardiovascular diseases were excluded from the current study.

### 2.2. Exposures

The exposure variable was salt intake. Participants were asked to rate their habitual salt intake as light, moderate, and salty. In addition, information on participants’ salt intake in the last 7 days was also collected and categorized into two levels: light to moderate and salty.

It has been reported that spot urinary sodium concentrations can reflect average 24 h urinary sodium concentrations and, therefore, could reflect salt intake at the population level, i.e., higher salt intake was associated with higher spot urinary concentrations [22]. Moreover, to examine the validity of the salt intake assessment, we collected random spot urine from 1324 participants and measured spot urinary sodium concentrations (mmol/L). There was a significantly positive association between salt intake and spot urinary sodium concentrations (*p* < 0.05), indicating the accuracy of the salt intake assessment in our study.

### 2.3. Outcomes

The outcome variables were three indicators of muscle strength and physical performance, i.e., grip strength (including absolute and relative grip strength (AGS and RGS)), the timed up-and-go test (TUGT), and falls in the past 6 months. Grip strength was tested by a Jamar Hydraulic Hand Dynamometer in a standing position. Grip strength assessment by the Jamar dynamometer showed good test-retest reproducibility (r > 0.80) [23] and excellent (r = 0.98) interrater reliability [24]. Each hand was measured two times, and the average value of each hand was calculated. The maximal value of the grip strength in left and right hands was used as the maximum absolute grip strength (AGS_max_), expressed as kilograms (kg). Maximum relative grip strength (RGS_max_) was equal to AGS_max_ divided by BMI. Average relative grip strength (RGS_mean_) was equal to the average value of the grip strength in both hands divided by BMI. Relative grip strength in the left- or right-hand (RGS_left_/RGS_right_) was equal to the grip strength in the left- or right-hand divided by BMI, respectively [25]. All the parameters of RGS were expressed as kg per kg/m^2^, as described in our previous studies [26,27,28]. 

The timed up-and-go test (TUGT) was tested by asking participants to get up from a chair, walk 2.5 m around a marker, and return. Nurses recorded the time used for the test for each participant. The test was assessed twice, and the scores (in seconds) were averaged. Detailed of the TUGT have been reported previously [27]. Information on self-reported falls was obtained by asking participants to recall how many times they have fallen in the past 6 months, and classified into two levels: having no falls, and ≥1 fall in the past 6 months. 

### 2.4. Potential Confounders

Potential confounders included sex, age (years), body mass index (BMI, kg/m^2^), education levels (primary or below, secondary, and college or above), occupation (manual, non-manual, and others), annually family income (<10,000, 10,000–29,999, 30,000–49,999, ≥50,000 CNY/year, and don’t know), smoking status (never, former, and current smokers), alcohol use (never, former, and current drinkers), physical activity (inactive, moderate, and active), self-rated health (good, and poor), and self-reported hypertension (no, and yes). We categorized alcohol use status into never, former, and current use, which has been described in our previous study [29]. Never drinkers were those who did not drink any alcoholic beverage throughout their life. Former drinkers were those who stopped drinking for more than one year. Current drinkers included occasional and regular drinkers, with occasional drinkers being those who drank less than once per week or only on special occasions, and regular drinkers being those who drank at least once per week. Physical activity was assessed using Chinese versions of the International Physical Activity Questionnaire (IPAQ-C) and categorized into inactive, moderate, and active [30]. We collected information on the frequency and duration of walking; all vigorous and moderate activities lasting at least 10 min; and time spent in sedentary activity. The reported minutes per week in each type of activity were weighted by the metabolic equivalent of the task (MET) based on its energy expenditure. Specifically, 1.0 MET was assigned for sitting, 3.3 METs for walking, 4.0 METs for moderate activity, and 8.0 METs for vigorous activity. The data were then converted to metabolic equivalent scores (MET-min/week) and physical activity levels were classified into 3 categories in ascending order (i.e., inactive, moderate, and active).

### 2.5. Statistical Analysis

Continuous variables were summarized as mean (standard deviation (SD)) or median (inter-quartile range (IQR)) as appropriate, and categorical variables were presented as numbers (percentages). Chi-square tests, one-way analysis of variance (ANOVA), and Kruskal-Wallis tests were used to compare participants’ basic characteristics by salt intake group. With the light salt intake group as the reference group, linear regression was used to assess the associations of general salt intake with the AGS, RGS, and TUGT scores, and logistic regression was used for falls in the past 6 months, giving regression coefficients (β) or odds ratios (OR) and 95% confidence intervals (CI) as appropriate. Furthermore, given the different levels of muscle strength and physical performance between men and women, we examined the interaction between general salt intake and sex on grip strength, the TUGT score, and falls and conducted stratification analysis by sex.

To assess the robustness of the results, we also performed analyses by assessing the associations of participants’ salt intake in the last 7 days with AGS, RGS, TUGT score, and falls. We also examined the interaction between general salt intake and potential moderators (occupation and age group (50–64/≥65 years)) on AGS, RGS, TUGT score, and falls, and also conducted stratification analysis by both of them. A sensitivity analysis excluding participants who reported poor self-rated health was used to assess whether the results varied by poor health status. We also assessed the association of spot urinary sodium concentrations with muscle strength and physical performance in 1324 participants. Stata version 16.0 (STATA Corp LP, TX, USA) and R program version 4.2.1 (ST Louis, MO, USA) were used for data analyses. All tests were two-sided, and *p* < 0.05 was considered statistically significant.

## 3. Results

Of the 6909 participants in the GBCS who completed physical function tests, 2042 were excluded due to incomplete information on salt intake or extreme values of muscle strength and physical performance indicators, leaving 4867 participants in the present study. The average age of the included participants was 60.4 (standard deviation (SD) = 7.7) years, and 72.2% were women. Of the 4867 participants, 19.0% reported poor self-rated health, 24.4% had self-reported hypertension, and 19.6% had antihypertensive drug use. Overall, the mean (SD) AGS_max_ and RGS_max_ were 25.2 (8.4) kg and 1.07 (0.38) kg per kg/m^2^, respectively. The median (IQR) TUGT score was 5.00 (0.95) seconds, and 6.3% of the participants reported ≥1 fall in the past 6 months. Regarding general salt intake levels, of the 4867 participants, 1925 (39.6%) had a light salt intake, 1789 (36.8%) had a moderate intake, and 1153 (23.6%) reported a salty taste in their daily life (Table 1). 

Table 1 shows that participants who were men with a higher BMI and had a lower education level and who were current smokers and current alcohol users tended to have salty foods (all *p* < 0.01). There was no significant association of salt intake with age, family income, occupation, physical activity, self-rated health, or serum sodium concentrations. The median (IQR) spot urinary sodium concentration across the three salt intake groups was 134.0 (93.0) mmol/L, 139.0 (92.0) mmol/L, and 144.0 (88.0) mmol/L, respectively, showing a significant difference in spot urinary sodium concentrations across the three salt intake groups (*p* < 0.05).

**Figure 1:**
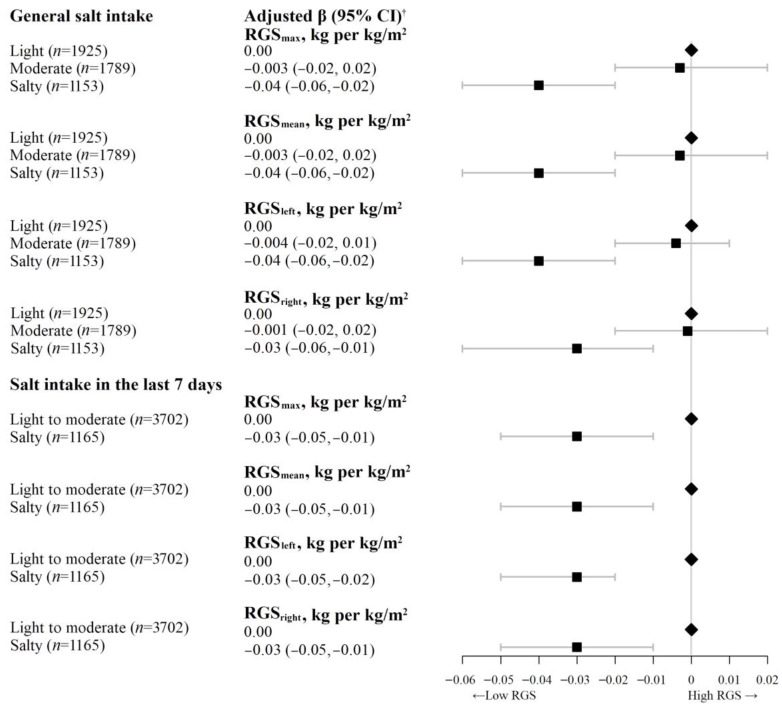
Association of salt intake with relative grip strength (kg per kg/m^2^) on 4867 participants of the Guangzhou Biobank Cohort Study. CI = confidence interval, RGS_max_ = maximum of the right or left relative grip strength, RGS_mean_ = average of the right and left relative grip strength, RGS_left_ = the left relative grip strength, RGS_right_ = the right relative grip strength. ^†^ Adjusted β (95% CI): adjusted for sex, age, education, family income, occupation, physical activity, smoking status, alcohol use, self-rated health, and self-reported hypertension.

Figure 1 shows that higher salt intake was significantly associated with lower RGS. After adjusting for sex, age, education, occupation, family income, smoking status, alcohol use, physical activity, self-rated health, and self-reported hypertension, compared with light salt intake, the RGS_max_, RGS_mean_, RGS_left_, and RGS_right_ in participants with salty taste decreased by 0.04, 0.04, 0.04, and 0.03 kg per kg/m^2^, respectively. The adjusted β (95% CI) was −0.04 (−0.06, −0.02) kg per kg/m^2^, −0.04 (−0.06, −0.02) kg per kg/m^2^, −0.04 (−0.06, −0.02) kg per kg/m^2^, and −0.03 (−0.06, −0.01) kg per kg/m^2^, respectively. Similar results were found between general salt intake and AGS_max_. Compared with light salt intake, those with salty taste had significantly lower AGS_max_ (β = −0.53 kg, 95% CI = −0.97 to −0.08, *p* < 0.05) (Figure S1).

**Figure 2:**
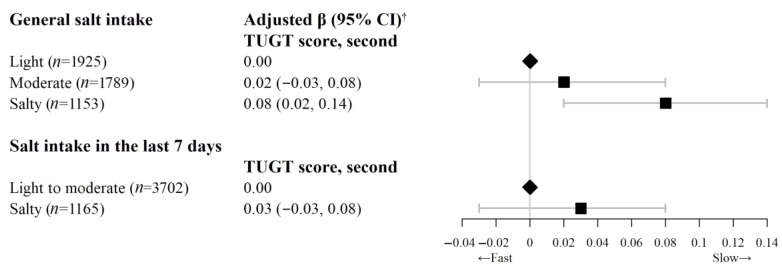
Association of salt intake with TUGT score (second) on 4867 participants of the Guangzhou Biobank Cohort Study. CI = confidence interval, TUGT = timed up-and-go test. ^†^ Adjusted β (95% CI): adjusted for sex, age, education, family income, occupation, physical activity, smoking status, alcohol use, body mass index (BMI), self-rated health, and self-reported hypertension.

**Figure 3:**
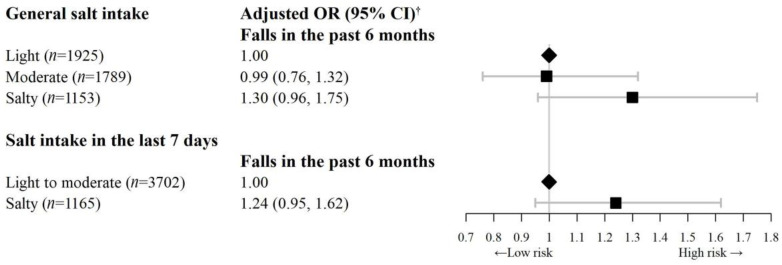
Association of salt intake with falls in the past 6 months on 4867 participants of the Guangzhou Biobank Cohort Study. OR = odds ratio, CI = confidence interval. ^†^ Adjusted OR (95% CI): adjusted for sex, age, education, family income, occupation, physical activity, smoking status, alcohol use, body mass index (BMI), self-rated health, and self-reported hypertension.

Figure 2 shows that after similar adjustment, compared with light salt intake, those with salty taste had significantly higher TUGT score (β = 0.08 s, 95% CI = 0.02 to 0.14, *p* < 0.05). Regarding falls in the past 6 months, compared with light salt intake, those with a salty taste had a marginally non-significantly higher risk of falls in the past 6 months (OR = 1.30, 95% CI = 0.96 to 1.75) (Figure 3).

No significant interaction was found between general salt intake and sex on grip strength, TUGT score, and falls (Table 2). When stratified by sex, the negative association between general salt intake and RGS remained significant in women, but became non-significant in men. Compared with light salt intake, the RGS in women with high salt intake decreased by 0.04 kg per kg/m^2^. The adjusted β (95% CI) of four indicators of RGS was −0.04 (−0.06, −0.02) kg per kg/m^2^ (*p* < 0.05, Table 2).

The associations of participants’ salt intake in the last 7 days with AGS, RGS, TUGT score, and falls showed the consistent results. Compared to light to moderate salt intake in the last 7 days, the RGS in participants with high salt intake decreased by 0.03 kg per kg/m^2^. The adjusted β (95% CI) of RGS_max_, RGS_mean_, RGS_left_, and RGS_right_ was −0.03 (−0.05, −0.01) kg per kg/m^2^, −0.03 (−0.05, −0.01) kg per kg/m^2^, −0.03 (−0.05, −0.02) kg per kg/m^2^, and −0.03 (−0.05, −0.01) kg per kg/m^2^, respectively (*p* < 0.05, Figure 1). Higher salt intake in the last 7 days was non-significantly associated with lower AGS, higher TUGT score and higher odds of falls in the past 6 months (Figure 2 and Figure 3, and Figure S1). No significant interaction was found between general salt intake and occupation on grip strength, TUGT score and falls (Table S1). Subgroup analysis by age group and sensitivity analysis excluding participants with poor self-rated health showed consistent results with those from the overall sample (Tables S2 and S3). No association between spot urinary sodium concentrations and AGS or RGS was found (Table S4).

## 4. Discussion

To our knowledge, this is the largest study to date reporting data concerning the association of salt intake with muscle strength as well as physical performance. We found that higher salt intake was significantly associated with poor upper-limb strength, poor balance performance, and lower gait speed, as indicated by lower hand grip strength and a higher TUGT score. The associations did not vary by sex or occupation. A generally consistent association was found for salt intake and falls in the past 6 months. As low muscle strength and physical performance are signs of frailty and an early manifestation of sarcopenia, our results provide important evidence to advocate for avoiding a high-salt diet in older people. 

Previous evidence on the association between salt intake and grip strength was relatively limited but showed consistent results [17,18]. A cross-sectional study on 114 Japanese reported that grip strength (i.e., absolute grip strength/body weight (BW)) of the high-salt group was significantly lower than that of the low-salt group (the mean (SD) grip strength was 0.46 (0.12) kg/BW and 0.54 (0.10) kg/BW, respectively, *p* < 0.05) [17]. However, this study showed the results without adjustment for potential confounders, and thus the estimates might be biased. Another cross-sectional study on 2982 older Korean participants also reported a higher risk of low grip strength (defined as AGS < 26 kg for men and <18 kg for women) in women with high sodium density (OR _Quartile 4 VS Quartile 2_ 1.51, 95% CI = 1.10 to 2.07), but not in men [18]. However, sodium intake and density were based on 24 h recall without validation by urine sodium. Habitual salt intake may reflect a more stable long-term diet habit than 24 h recall. Since grip strength was strongly correlated with body size [31,32], RGS was more commonly used as a proxy for muscle strength with adjustment of BMI [33]. Therefore, we included various parameters of grip strength (including AGS_max_, RGS_max_, RGS_mean_, RGS_left_, and RGS_right_) in our analysis and found consistently negative associations of salt intake with all parameters of grip strength in the overall sample and in women but not in men, although the non-significant results in men may be partly explained by the small sample size. Our results thus support findings from previous studies and add to the literature by providing robust estimates using a larger sample size with reliable measures of grip strength. Moreover, previous studies based on MRI imaging reported an increase of sodium signal in muscle during normal aging, indicating an increasing sodium concentration or an enlarged extracellular matrix (ECM) volume fraction [34]. Given sodium deposition in skeletal muscle tissues in the elderly may be higher than that of young people [12,34], our findings provide useful epidemiologic information for advocating salt reduction in older people to prevent muscle strength decline.

TUGT was widely used to assess physical performance, especially balance performance, gait speed, functional capacity, and lower limb strength in older people [20,35]. Our study for the first time found the positive correlation between salt intake and TUGT score. The associations of salt intake with different measures of physical performance reported in previous studies have been inconclusive [14,17]. For example, a study of 114 Japanese reported that salt intake was significantly negatively associated with performance in lower limb strength evaluated by the chair rise test but not with gait speed or balance performance assessed by single-leg stance time (SLT) [17]. Another study based on the Seniors-ENRICA cohort reported that, compared with those with a 5-year minimal change in sodium intake, participants with a 5-year increase in dietary sodium intake had significantly worse physical performance, as indicated by items related to the chair stand and gait speed tests, but not a difference in balance performance [14]. The discrepancy may be partly explained by the various measurements used in different studies. With simple steps and no special equipment required, TUGT is a quick and reliable measure to integrally evaluate physical mobility, including balance, gait, and lower limb strength in older people [20]. The positive correlation between salt intake and TUGT score in our analysis indicated a potentially detrimental role of salt intake on physical performance, specifically poor balance performance, poor lower limb strength, and slow gait speed, even after adjusting for multiple confounding factors. Further prospective studies exploring the impact of salt intake on sarcopenia or frailty are warranted.

Although higher salt intake was significantly associated with lower grip strength and a higher TUGT score, the association of salt intake with falls in our analysis was non-significant. Guideline for the Prevention of Falls in Older Persons has summarized multiple independent risk factors of falls, including low muscle strength and balance deficits [36]. Although several previous studies and ours reported the inverse association of salt intake with muscle strength [17,18], existing evidence showed no association between salt intake and balance deficit [14,17]. To note, a meta-analysis reported a weak association between balance and lower limb strength, which was probably due to the independent neuromuscular components of each other [37]. Given the lack of prospective evidence and the small sample size of participants reporting falls in our analysis, further prospective studies with a larger sample size are warranted to confirm the association between salt intake and falls.

Several mechanisms could explain the adverse effects of high salt intake on muscle strength and physical performance. One of the explanations was that salty taste, accompanied by high sodium intake, would lead to excessive potassium excretion and a high Na^+^/K^+^ ratio [38]. It was reported that high a Na^+^/K^+^ ratio was associated with high blood pressure and low peripheral blood volume [39]. As a sufficient peripheral blood supply is required to maintain adequate muscle strength via the activation of the energy sensor AMPK [40], abnormal potassium homeostasis and a high Na^+^/K^+^ ratio may cause low peripheral blood supply and subsequently reduce the energy supplied to muscle contraction [41]. This would lead to poor muscle strength and physical performance. Besides, high salt intake was also related to low calcium levels, with detrimental effects on bone health and physical performance, which was supported by a previous study showing that high salt intake accelerated bone turnover and increased urinary calcium excretion [42]. 

The strengths of our study included the population-based design, the use of reliable measures for muscle strength and physical performance, and the detailed assessment of potential confounders. However, there were several limitations in our study. First, information on salt intake was collected via self-reported and subjective questions, a method prone to measurement error. However, we compared the spot urinary sodium concentration levels across three salt intake groups and found higher spot urinary sodium concentrations in those with salty tastes, indicating the accuracy of the subjective salt intake report in our study. Second, our analysis may not be adequately powered to assess the association of salt intake with falls risk due to the relatively small proportion of participants reporting falls in the past 6 months. Moreover, the sample size may also be insufficient for conducting subgroup analyses by sex or assessing the association of spot urinary sodium concentrations with grip strength, timed up-and-go test and falls, given that the effect sizes of the associations are likely to be small. Third, although multiple potential confounders were included, residual confounding due to unmeasured factors such as total energy intake also remained in our analysis. However, compared with objective measurements of salt intake, there was a relatively weak correlation between the subjective salt report and total energy intake [43]. Moreover, no consensus on the adjustment of total energy intake in epidemiologic studies has been reached [44]. Fourth, the causal association of salt intake with muscle strength, and physical performance could not be confirmed in this cross-sectional study. However, as older Chinese had a relatively stable taste preference, general salt intake may reflect a long-term diet habit, and thus, the temporality was unlikely to be reversed.

## 5. Conclusions

In conclusion, we found that higher salt intake was significantly associated with lower grip strength and a higher TUGT score, indicating the potentially detrimental role of high-salt diet in muscle strength and physical performance in older Chinese, especially in older women. The causal relationship between salt intake and muscle strength as well as physical performance needs to be confirmed in further experimental studies.

## Figures and Tables

**Table 1:** Characteristics by salt intake on 4867 participants of the Guangzhou Biobank Cohort Study.

	All	Salt Intake			*P* Value
		Light	Moderate	Salty	
Number of participants, *N* (%)	4867 (100.0)	1925 (39.6)	1789 (36.8)	1153 (23.6)	-
Men, %	27.8	21.7	30.0	34.4	<0.01
Age, years, mean (SD)	60.4 (7.7)	60.2 (7.6)	60.6 (7.7)	60.5 (7.8)	0.31
Family income, CNY/year, %					0.15
<10,000	4.6	4.1	4.8	5.2	
10,000–29,999	31.6	32.6	31.3	30.7	
30,000–49,999	27.1	26.9	27.3	27.1	
≥50,000	21.9	22.4	20.2	23.4	
Don’t know	14.8	14.0	16.4	13.6	
Education, %					<0.01
Primary or below	36.1	32.7	38.0	38.8	
Secondary	54.8	56.5	53.7	53.6	
College or above	9.1	10.8	8.3	7.6	
Occupation, %					0.29
Manual	61.3	59.5	62.8	61.7	
Non-manual	20.7	22.0	19.7	20.0	
Others	18.0	18.5	17.5	18.3	
Smoking status, %					<0.01
Never	80.4	85.9	78.8	74.0	
Former	8.3	6.7	8.6	10.8	
Current	11.3	7.4	12.6	15.2	
Alcohol use, %					0.01
Never	35.2	37.8	34.0	32.6	
Former	3.7	3.8	4.0	3.1	
Current	61.1	58.4	62.0	64.3	
Physical activity, %					0.07
Inactive	5.4	5.4	4.5	6.9	
Moderate	30.5	29.7	31.5	30.2	
Active	64.1	64.9	64.0	62.9	
Self-rated health, %					0.39
Good	81.0	81.6	81.3	79.6	
Poor	19.0	18.4	18.7	20.4	
Self-reported hypertension, %					0.02
No	75.6	73.7	77.6	75.7	
Yes	24.4	26.3	22.4	24.3	
Antihypertensive drugs use, %					
No	80.4	77.8	82.5	81.7	<0.01
Yes	19.6	22.2	17.5	18.3	
Falls in the past 6 months, %	6.3	6.1	5.8	7.3	0.25
BMI, kg/m^2^, mean (SD)	23.9 (3.3)	23.7 (3.2)	23.9 (3.3)	24.2 (3.4)	<0.01
AGS_max_, kg, mean (SD)	25.2 (8.4)	24.4 (7.9)	25.7 (8.6)	25.6 (8.6)	<0.01
RGS_max_, kg per kg/m^2^, mean (SD)	1.07 (0.38)	1.05 (0.36)	1.09 (0.39)	1.08 (0.39)	<0.01
RGS_mean_, kg per kg/m^2^, mean (SD)	1.04 (0.37)	1.02 (0.35)	1.06 (0.38)	1.05 (0.39)	<0.01
RGS_left_, kg per kg/m^2^, mean (SD)	1.05 (0.38)	1.03 (0.36)	1.07 (0.39)	1.05 (0.39)	<0.01
RGS_right_, kg per kg/m^2^, mean (SD)	1.03 (0.37)	1.00 (0.35)	1.05 (0.38)	1.04 (0.39)	<0.01
TUGT score, second, median (IQR)	5.00 (0.95)	4.97 (0.93)	5.02 (0.97)	5.06 (1.02)	0.02
Serum sodium concentration, mmol/L, median (IQR)	145.0 (4.0)	145.0 (4.0)	145.0 (4.0)	145.0 (4.0)	0.56
Urinary sodium concentration, mmol/L, median (IQR)	136.0 (92.0)	134.0 (93.0)	139.0 (92.0)	144.0 (88.0)	0.02

SD = standard deviation, IQR = inter-quartile range, BMI = body mass index, AGS_max_ = maximum of the right or left absolute grip strength, RGS_max_ = maximum of the right or left relative grip strength, RGS_mean_ = average of the right and left relative grip strength, RGS_left_ = the left relative grip strength, RGS_right_ = the right relative grip strength, TUGT = timed up-and-go test, CNY = Chinese yuan. Serum sodium concentration (mmol/L) and urinary sodium concentration (mmol/L) was measured in 1330 and 1324 participants, respectively.

**Table 2:** Associations of general salt intake with grip strength, timed up-and-go test, and falls in 1351 men and 3516 women of the Guangzhou Biobank Cohort Study.

	Men			Women			P for Interaction
	Light	Moderate	Salty	Light	Moderate	Salty	
AGS_max_, kg							0.68
Crude β (95% CI)	0.00	0.52 (−0.52, 1.57)	−0.71 (−1.84, 0.41)	0.00	0.18 (−0.24, 0.60)	−0.17 (−0.66, 0.32)	
Adjusted β (95% CI) ^ξ^	0.00	0.28 (−0.68, 1.23)	−0.61 (−1.64, 0.41)	0.00	0.18 (−0.22, 0.59)	−0.40 (−0.87, 0.08)	
RGS_max_, kg per kg/m^2^							0.85
Crude β (95% CI)	0.00	0.02 (−0.03, 0.07)	−0.03 (−0.08, 0.02)	0.00	−0.01 (−0.03, 0.01)	−0.04 (−0.06, −0.02) **	
Adjusted β (95% CI) ^ξ^	0.00	0.002 (−0.04, 0.05)	−0.03 (−0.08, 0.02)	0.00	−0.01 (−0.02, 0.01)	−0.04 (−0.06, −0.02) ***	
RGS_mean_, kg per kg/m^2^							0.90
Crude β (95% CI)	0.00	0.02 (−0.03, 0.06)	−0.03 (−0.08, 0.02)	0.00	−0.01 (−0.03, 0.01)	−0.04 (−0.06, −0.01) **	
Adjusted β (95% CI) ^ξ^	0.00	−0.0001 (−0.04, 0.04)	−0.03 (−0.08, 0.02)	0.00	−0.005 (−0.02, 0.01)	−0.04 (−0.06, −0.02) ***	
RGS_left_, kg per kg/m^2^							0.76
Crude β (95% CI)	0.00	0.02 (−0.03, 0.07)	−0.04 (−0.09, 0.01)	0.00	−0.01 (−0.03, 0.01)	−0.04 (−0.06, −0.02) **	
Adjusted β (95% CI) ^ξ^	0.00	0.002 (−0.04, 0.05)	−0.04 (−0.09, 0.01)	0.00	−0.01 (−0.03, 0.01)	−0.04 (−0.06, −0.02) ***	
RGS_right_, kg per kg/m^2^							0.82
Crude β (95% CI)	0.00	0.02 (−0.03, 0.06)	−0.02 (−0.07, 0.03)	0.00	−0.01 (−0.02, 0.01)	−0.03 (−0.06, −0.01) **	
Adjusted β (95% CI) ^ξ^	0.00	−0.002 (−0.05, 0.04)	−0.02 (−0.07, 0.03)	0.00	−0.002 (−0.02, 0.02)	−0.04 (−0.06, −0.02) **	
TUGT score, second							0.84
Crude β (95% CI)	0.00	−0.01 (−0.14, 0.12)	0.12 (−0.02, 0.26)	0.00	0.08 (0.01, 0.16) *	0.10 (0.01, 0.19) *	
Adjusted β (95% CI) ^ξ^	0.00	0.01 (−0.11, 0.12)	0.09 (−0.03, 0.22)	0.00	0.03 (−0.04, 0.09)	0.07 (−0.002, 0.15)	
Falls in the past 6 months							0.79
Crude OR (95% CI)	1.00	0.87 (0.44, 1.69)	1.38 (0.72, 2.65)	1.00	1.02 (0.76, 1.38)	1.26 (0.90, 1.75)	
Adjusted OR (95% CI) ^ξ^	1.00	0.79 (0.40, 1.56)	1.26 (0.65, 2.47)	1.00	1.03 (0.76, 1.39)	1.27 (0.91, 1.78)	

CI = confidence interval, OR=odds ratio, AGS_max_ = maximum of the right or left absolute grip strength, RGS_max_ = maximum of the right or left relative grip strength, RGS_mean_ = average of the right and left relative grip strength, RGS_left_ = the left relative grip strength, RGS_right_ = the right relative grip strength, TUGT = timed up-and-go test. ^ξ^ Adjusted for age, education, family income, occupation, physical activity, smoking status, alcohol use, body mass index (BMI, except for RGS), self-rated health, and self-reported hypertension. * *p* < 0.05, ** *p* < 0.01, *** *p* < 0.001.

## Data Availability

Not applicable.

## Supplementary Materials

The following supporting information can be downloaded at: https://www.mdpi.com/article/10.3390/nu15030516/s1, Figure S1: Association of salt intake with absolute grip strength (kg) on 4867 participants of the Guangzhou Biobank Cohort Study. Table S1: Associations of general salt intake with grip strength, timed up-and-go test, and falls in 2981 manual workers and 1886 non-manual workers of the Guangzhou Biobank Cohort Study. Table S2: Associations of general salt intake with grip strength, timed up-and-go test, and falls in 3571 middle-aged participants (50–64 years) and 1296 older participants (≥65 years) of the Guangzhou Biobank Cohort Study. Table S3: Associations of general salt intake with grip strength, timed up-and-go test, and falls in 3942 participants with good self-rated health of the Guangzhou Biobank Cohort Study. Table S4: Associations of spot urinary sodium concentrations (mmol/L) with grip strength, timed up-and-go test, and falls on 1324 participants of the Guangzhou Biobank Cohort Study.
