# Supplementary material for: Association of Salt Intake with Muscle Strength and Physical Performance in Middle-Aged to Older Chinese: The Guangzhou Biobank Cohort Study
Source: Nutrients. 2023 Jan 19;15(3):516. doi: 10.3390/nu15030516 (PMC9919999; doi:10.3390/nu15030516)
Supplement: Supplementary file 1 [file nutrients-15-00516-s001.zip › nutrients-2155204-supplementary.pdf]

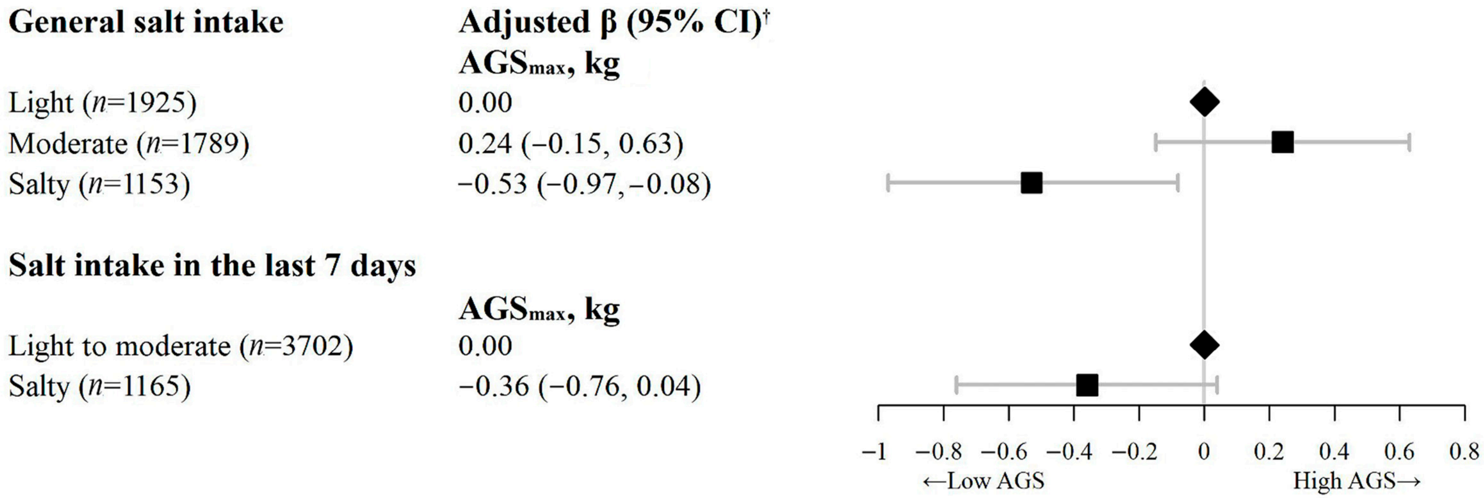

Figure S1 Association of salt intake with absolute grip strength (kg) on 4867 participants of the Guangzhou Biobank Cohort Study

CI=confidence interval, AGS_{max}=maximum of the right or left absolute grip strength.

†Adjusted β (95% CI): adjusted for sex, age, education, family income, occupation, physical activity, smoking status, alcohol use, body mass index (BMI), self-rated health, and self-reported hypertension.–

Table S1 Associations of general salt intake with grip strength, timed up-and-go test, and falls in 2981 manual workers and 1886 non-manual workers of the Guangzhou Biobank Cohort Study

	Manual workers			Non-manual workers			*P* for interaction
	Light	Moderate	Salty	Light	Moderate	Salty	
AGS_{max}, kg							0.65
Crude β (95% CI)	0.00	0.96 (0.30, 1.62) **	1.06 (0.30, 1.81) **	0.00	1.92 (1.02, 2.82) ***	1.42 (0.41, 2.43) **	
Adjusted β (95% CI) ξ	0.00	0.27 (−0.22, 0.76)	−0.37 (−0.93, 0.19)	0.00	0.18 (−0.47, 0.82)	−0.78 (−1.52, −0.05) *	
RGS_{max}, kg per kg/m^2							0.92
Crude β (95% CI)	0.00	0.03 (−0.005, 0.06)	0.02 (−0.01, 0.05)	0.00	0.08 (0.04, 0.12) ***	0.05 (0.004, 0.09) *	
Adjusted β (95% CI) ξ	0.00	−0.005 (−0.03, 0.02)	−0.04 (−0.07, −0.01) **	0.00	0.00 (−0.03, 0.03)	−0.04 (−0.08, −0.01) *	
RGS_{mean}, kg per kg/m^2							0.93
Crude β (95% CI)	0.00	0.02 (−0.01, 0.05)	0.02 (−0.01, 0.05)	0.00	0.08 (0.04, 0.11) ***	0.05 (0.01, 0.10) *	
Adjusted β (95% CI) ξ	0.00	−0.01 (−0.03, 0.02)	−0.04 (−0.07, −0.01) **	0.00	0.002 (−0.03, 0.03)	−0.04 (−0.07, −0.005) *	
RGS_{left}, kg per kg/m^2							0.88
Crude β (95% CI)	0.00	0.02 (−0.01, 0.05)	0.02 (−0.02, 0.05)	0.00	0.07 (0.04, 0.11) ***	0.05 (0.001, 0.09) *	
Adjusted β (95% CI) ξ	0.00	−0.01 (−0.03, 0.02)	−0.04 (−0.07, −0.02) **	0.00	0.001 (−0.03, 0.03)	−0.04 (−0.08, −0.01) *	
RGS_{right}, kg per kg/m^2							0.96
Crude β (95% CI)	0.00	0.03 (−0.004, 0.06)	0.02 (−0.01, 0.06)	0.00	0.08 (0.04, 0.11) ***	0.06 (0.01, 0.10) *	
Adjusted β (95% CI) ξ	0.00	−0.004 (−0.03, 0.02)	−0.04 (−0.06, −0.01) **	0.00	0.002 (−0.03, 0.03)	−0.03 (−0.07, 0.001)	
TUGT score, second							0.09
Crude β (95% CI)	0.00	0.12 (0.03, 0.20) **	0.12 (0.03, 0.22) *	0.00	−0.06 (−0.16, 0.04)	0.08 (−0.03, 0.19)	
Adjusted β (95% CI) ξ	0.00	0.07 (0.002, 0.15) *	0.12 (0.03, 0.20) **	0.00	−0.05 (−0.14, 0.03)	0.02 (−0.08, 0.12)	
Falls in the past 6 months							0.73
Crude OR (95% CI)	1.00	1.07 (0.75, 1.53)	1.25 (0.85, 1.84)	1.00	0.81 (0.52, 1.24)	1.18 (0.76, 1.84)	
Adjusted OR (95% CI) ξ	1.00	1.09 (0.76, 1.57)	1.36 (0.92, 2.01)	1.00	0.85 (0.55, 1.33)	1.23 (0.77, 1.95)	

CI=confidence interval, OR=odds ratio, AGS_{max}=maximum of the right or left absolute grip strength, RGS_{max}=maximum of the right or left relative grip strength, RGS_{mean}=average of the right

and left relative grip strength, RGS_{left}=the left relative grip strength, RGS_{right}=the right relative grip strength, TUGT=timed up-and-go test.

[ξ]Adjusted for sex, age, education, family income, physical activity, smoking status, alcohol use, body mass index (BMI, except for RGS), self-rated health, and self-reported hypertension.

[*]P<0.05, [**]P<0.01, [***]P<0.001.

Table S2 Associations of general salt intake with grip strength, timed up-and-go test, and falls in 3571 middle-aged participants (50-64 years) and 1296 older participants (≥65 years) of the Guangzhou Biobank Cohort Study

	Middle-aged participants (50-64 years)			Older participants (≥65 years)			*P* for interaction
	Light	Moderate	Salty	Light	Moderate	Salty	
AGS_{max}, kg							0.36
Crude β (95% CI)	0.00	1.57 (0.94, 2.19) [***]	1.09 (0.37, 1.80) [**]	0.00	0.62 (−0.40, 1.64)	1.49 (0.34, 2.65) [*]	
Adjusted β (95% CI) [ξ]	0.00	0.36 (−0.11, 0.82)	−0.32 (−0.85, 0.21)	0.00	0.05 (−0.70, 0.81)	−0.57 (−1.44, 0.30)	
RGS_{max}, kg per kg/m^2							0.43
Crude β (95% CI)	0.00	0.05 (0.02, 0.08) [***]	0.03 (−0.01, 0.06)	0.00	0.02 (−0.02, 0.07)	0.05 (−0.01, 0.10)	
Adjusted β (95% CI) [ξ]	0.00	0.001 (−0.02, 0.02)	−0.03 (−0.06, −0.01) [*]	0.00	−0.01 (−0.04, 0.03)	−0.05 (−0.09, −0.01) [*]	
RGS_{mean}, kg per kg/m^2							0.46
Crude β (95% CI)	0.00	0.05 (0.02, 0.08) [***]	0.03 (−0.01, 0.06)	0.00	0.02 (−0.02, 0.07)	0.05 (−0.01, 0.10)	
Adjusted β (95% CI) [ξ]	0.00	0.00002 (−0.02, 0.02)	−0.03 (−0.05, −0.01) [*]	0.00	−0.01 (−0.04, 0.03)	−0.05 (−0.09, −0.01) [*]	
RGS_{left}, kg per kg/m^2							0.53
Crude β (95% CI)	0.00	0.05 (0.02, 0.08) [**]	0.02 (−0.01, 0.05)	0.00	0.03 (−0.02, 0.07)	0.05 (−0.01, 0.10)	
Adjusted β (95% CI) [ξ]	0.00	−0.003 (−0.02, 0.02)	−0.03 (−0.06, −0.01) [**]	0.00	−0.004 (−0.04, 0.03)	−0.05 (−0.09, −0.01) [*]	
RGS_{right}, kg per kg/m^2							0.40
Crude β (95% CI)	0.00	0.05 (0.02, 0.08) [***]	0.03 (−0.001, 0.06)	0.00	0.02 (−0.02, 0.07)	0.05 (−0.004, 0.10)	
Adjusted β (95% CI) [ξ]	0.00	0.003 (−0.02, 0.02)	−0.02 (−0.05, −0.0001) [*]	0.00	−0.01 (−0.04, 0.03)	−0.04 (−0.08, −0.003) [*]	
TUGT score, second							0.24
Crude β (95% CI)	0.00	0.02 (−0.03, 0.07)	0.07 (0.01, 0.13) [*]	0.00	0.09 (−0.08, 0.25)	0.15 (−0.04, 0.34)	
Adjusted β (95% CI) [ξ]	0.00	−0.004 (−0.06, 0.05)	0.03 (−0.03, 0.09)	0.00	0.07 (−0.09, 0.23)	0.17 (−0.01, 0.36)	

Falls in the past 6 months							0.92
Crude OR (95% CI)	1.00	0.98 (0.70, 1.36)	1.22 (0.85, 1.74)	1.00	0.89 (0.55, 1.43)	1.18 (0.72, 1.96)	
Adjusted OR (95% CI) [ξ]	1.00	1.02 (0.73, 1.44)	1.26 (0.87, 1.81)	1.00	0.94 (0.58, 1.52)	1.34 (0.79, 2.25)	

CI=confidence interval, OR=odds ratio, AGS_{max}=maximum of the right or left absolute grip strength, RGS_{max}=maximum of the right or left relative grip strength, RGS_{mean}=average of the right and left relative grip strength, RGS_{left}=the left relative grip strength, RGS_{right}=the right relative grip strength, TUGT=timed up-and-go test.

[ξ]Adjusted for sex, education, family income, occupation, physical activity, smoking status, alcohol use, body mass index (BMI, except for RGS), self-rated health, and self-reported hypertension.

$^{*}P<0.05$, $^{**}P<0.01$, $^{***}P<0.001$.

Table S3 Associations of general salt intake with grip strength, timed up-and-go test, and falls in 3942 participants with good self-rated health of the Guangzhou Biobank Cohort Study

	Participants with good self-rated health		
	Light	Moderate	Salty
AGS_{max}, kg			
Crude β (95% CI)	0.00	1.24 (0.63, 1.84) ***	1.29 (0.60, 1.97) ***
Adjusted β (95% CI) ξ	0.00	0.35 (−0.10, 0.81)	−0.31 (−0.83, 0.22)
RGS_{max}, kg per kg/m^2			
Crude β (95% CI)	0.00	0.04 (0.02, 0.07) **	0.04 (0.005, 0.07) *
Adjusted β (95% CI) ξ	0.00	0.004 (−0.02, 0.02)	−0.03 (−0.06, −0.01) *
RGS_{mean}, kg per kg/m^2			
Crude β (95% CI)	0.00	0.04 (0.02, 0.07) **	0.04 (0.01, 0.07) *
Adjusted β (95% CI) ξ	0.00	0.004 (−0.02, 0.02)	−0.03 (−0.05, −0.01) *
RGS_{left}, kg per kg/m^2			
Crude β (95% CI)	0.00	0.04 (0.02, 0.07) **	0.03 (0.001, 0.06) *
Adjusted β (95% CI) ξ	0.00	0.004 (−0.02, 0.02)	−0.04 (−0.06, −0.01) **
RGS_{right}, kg per kg/m^2			
Crude β (95% CI)	0.00	0.04 (0.02, 0.07) **	0.04 (0.01, 0.07) *
Adjusted β (95% CI) ξ	0.00	0.004 (−0.02, 0.02)	−0.03 (−0.05, −0.002) *
TUGT score, second			
Crude β (95% CI)	0.00	0.05 (−0.02, 0.12)	0.08 (0.01, 0.16) *
Adjusted β (95% CI) ξ	0.00	0.01 (−0.06, 0.07)	0.03 (−0.04, 0.11)
Falls in the past 6 months			
Crude OR (95% CI)	1.00	1.02 (0.74, 1.40)	1.19 (0.84, 1.68)
Adjusted OR (95% CI) ξ	1.00	1.07 (0.78, 1.48)	1.30 (0.91, 1.85)

CI=confidence interval, OR=odds ratio, AGS_{max}=maximum of the right or left absolute grip strength, RGS_{max}=maximum of the right or left relative grip strength, RGS_{mean}=average of the right and left relative grip strength, RGS_{left}=the left relative grip strength, RGS_{right}=the right relative grip strength, TUGT=timed up-and-go test.

ξAdjusted for sex, age, education, family income, occupation, physical activity, smoking status, alcohol use, body mass index (BMI, except for RGS), and self-reported hypertension.

*P<0.05, **P<0.01, ***P<0.001.

Table S4 Associations of spot urinary sodium concentrations (mmol/L) with grip strength, timed up-and-go test, and falls on 1324 participants of the Guangzhou Biobank Cohort Study

	Spot urinary sodium concentrations (mmol/L)			
	Quartile 1 (n=342)	Quartile 2 (n=330)	Quartile 3 (n=313)	Quartile 4 (n=339)
AGS_{max}, kg				
Crude β (95% CI)	0.00	−0.26 (−1.67, 1.16)	0.67 (−0.77, 2.10)	1.92 (0.51, 3.33) **
Adjusted β (95% CI) ξ	0.00	−0.73 (−1.77, 0.31)	−0.27 (−1.33, 0.79)	0.98 (−0.07, 2.02)
RGS_{max}, kg per kg/m^2				
Crude β (95% CI)	0.00	−0.001 (−0.06, 0.06)	0.02 (−0.04, 0.08)	0.08 (0.02, 0.15) *
Adjusted β (95% CI) ξ	0.00	−0.03 (−0.07, 0.02)	−0.02 (−0.07, 0.03)	0.04 (−0.01, 0.08)

RGS_{mean}, kg per kg/m^2				
Crude β (95% CI)	0.00	−0.002 (−0.06, 0.06)	0.02 (−0.04, 0.08)	0.08 (0.02, 0.14)
Adjusted β (95% CI) $^{\xi}$	0.00	−0.03 (−0.07, 0.02)	−0.02 (−0.07, 0.03)	0.04 (−0.01, 0.08)
RGS_{left}, kg per kg/m^2				
Crude β (95% CI)	0.00	−0.0004 (−0.06, 0.06)	0.02 (−0.05, 0.08)	0.08 (0.02, 0.14)
Adjusted β (95% CI) $^{\xi}$	0.00	−0.03 (−0.07, 0.02)	−0.02 (−0.07, 0.03)	0.03 (−0.01,0.08)
RGS_{right}, kg per kg/m^2				
Crude β (95% CI)	0.00	−0.003 (−0.06, 0.06)	0.02 (−0.04, 0.09)	0.08 (0.02, 0.15) **
Adjusted β (95% CI) $^{\xi}$	0.00	−0.03 (−0.08, 0.02)	−0.01 (−0.06, 0.04)	0.04 (−0.01, 0.09)
TUGT score, second				
Crude β (95% CI)	0.00	−0.03 (−0.14, 0.08)	−0.04 (−0.15, 0.07)	−0.03 (−0.14, 0.08)
Adjusted β (95% CI) $^{\xi}$	0.00	−0.05 (−0.14, 0.05)	−0.05 (−0.15, 0.05)	−0.004 (−0.10, 0.09)
Falls in the past 6 months				
Crude OR (95% CI)	1.00	0.87 (0.44, 1.71)	0.74 (0.36, 1.52)	0.73 (0.36, 1.49)
Adjusted OR (95% CI) $^{\xi}$	1.00	0.86 (0.43, 1.72)	0.73 (0.35, 1.52)	0.77 (0.37, 1.59)

CI=confidence interval, OR=odds ratio, AGS_{max}=maximum of the right or left absolute grip strength, RGS_{max}=maximum of the right or left relative grip strength, RGS_{mean}=average of the right and left relative grip strength, RGS_{left}=the left relative grip strength, RGS_{right}=the right relative grip strength, TUGT=timed up-and-go test.

$^{\xi}$Adjusted for sex, age, education, family income, occupation, physical activity, smoking status, alcohol use, body mass index (BMI, except for RGS), systolic blood pressure (SBP), diastolic blood pressure (DBP), and antihypertensive drugs use.

$^{*}P<0.05$, $^{**}P<0.01$, $^{***}P<0.001$.
